# Gestational influenza and risk of hypomania in young adulthood: prospective birth cohort study

**DOI:** 10.1016/j.jad.2016.04.048

**Published:** 2016-08

**Authors:** Jana J. Anderson, Sean Hoath, Stanley Zammit, Thomas D. Meyer, Jill P Pell, Daniel Mackay, Daniel J. Smith

**Affiliations:** a Institute of Health & Wellbeing, University of Glasgow, 1 Lilybank Gardens, G12 8RZ Glasgow, Scotland, UK; b Department of Psychological Medicine and Clinical Neurosciences, School of Medicine, Cardiff University, Wales, UK; c Department of Psychiatry and Behavioral Sciences, University of Texas Health Science Center at Houston, TX, USA

**Keywords:** PE, Psychotic experiences, Influenza, Pregnancy, Bipolar disorder, Psychotic disorders, ALSPAC

## Abstract

**Background:**

Previous studies have suggested a possible link between exposure to influenza in utero and bipolar disorder in adulthood. Using data from a prospective birth cohort, we aimed to test for an association between exposure to gestational influenza and the experience of hypomania assessed in early adulthood.

**Methods:**

We used data on 2957 participants from the Avon Longitudinal Study of Parents and Children (ALSPAC). The two main outcomes of interest were hypomania, assessed using the Hypomania Checklist (HCL-32) at age 22–23, and ‘hypomania plus previous psychotic experiences (PE)’. Maternally-reported gestational influenza was the exposure of interest. Multivariable logistic regression was used and estimates of association were adjusted for a range of possible confounding factors, including maternal smoking in pregnancy.

**Results:**

Relative to controls, rates of exposure to gestational influenza were higher for participants with hypomania (24.0%) and for participants with ‘hypomania plus PE’ (34.2%), but univariate and multivariable analyses of an association between gestational influenza and hypomania (with and without previous PE) were not significant.

**Limitations:**

The response rate to those who were sent the HCL-32 questionnaire was 36.8%. As a result, some analyses may have been under-powered to detect a true effect. Influenza infection during pregnancy was self-reported by mothers.

**Conclusions:**

In this prospective population study, gestational influenza was not identified as a clear risk factor for lifetime hypomania or for ‘hypomania with PEs’ in young adult offspring. It is possible that previous reports of an association between gestational influenza and bipolar disorder in adulthood have been confounded by factors such as maternal smoking during pregnancy.

## Highlights

• Prospective birth cohort study controlling for wide range of potential confounders.
• Rates of gestational influenza were higher for hypomania groups than controls.
• However, associations not significant when adjusted for confounders.
• Future studies should adjust for confounders, such as maternal smoking.

## 1 Introduction

Bipolar disorder (BD) affects at least 2% of the global population and is characterised by episodes of depression alternating with episodes of mania or hypomania (Goodwin and Jamison, 2007). It is associated with significant psychological, social, occupational and physical health morbidity, as well as premature mortality due to suicide and cardiometabolic disease (Goodwin and Jamison, 2007, Walker et al., 2015). Our current understanding of the etiology of BD suggests that it occurs as a consequence of complex interactions between multiple genetic risk factors of small effect and non-genetic factors such as childhood trauma, adverse life events and substance misuse (Garno et al., 2005, Phillips and Kupfer, 2013). In recent years, autoimmunity and aberrant inflammatory processes have also been implicated in the pathophysiology of BD, alongside other mood and psychotic disorders such as major depression and schizophrenia (Berk et al., 2013, Dickerson et al., 2007, Frommberger et al., 1997, Harrison, 2013, Khandaker et al., 2015, Krishnadas and Cavanagh, 2012). One of the potential mechanisms of the association between neuroinflammation and psychiatric disorders might be infection in utero acting as a trigger for a cascade of abnormal immune responses during early development (Berk et al., 2013, Khandaker et al., 2012, Khandaker et al., 2013).

Recent work suggests that gestational influenza (infection occurring during pregnancy) may be a risk factor for BD in adult offspring, however, not all studies have found an association (Simanek and Meier, 2015). Parboosing and colleagues conducted a nested case-control analysis of 92 BD cases and 722 controls within the Child Health and Development Study (CHDS) in the United States (Parboosing et al., 2013). They reported an association between self-reported gestational influenza and BD with and without psychotic symptoms, which appeared to be specific to influenza exposure during the third trimester. However, a subsequent analysis of the same sample, which this time used serologically-documented gestational influenza as the exposure of interest, did not find an association with BD in offspring, but did find that gestational influenza increased the risk of BD with psychotic features (Canetta et al., 2014). Alongside evidence that gestational influenza may be a risk factor for schizophrenia in offspring (Brown and Derkits, 2010), this suggests that prenatal exposure to influenza may be a non-specific risk factor for psychosis or severe psychopathology, rather than specific for a diagnostic grouping such as schizophrenia or BD.

Additionally, maternal smoking during pregnancy is known to be associated with psychotic experiences during adolescence (Zammit et al., 2009b) and schizophrenia in adulthood (Stathopoulou et al., 2013). Although the CHDS studies cited above adjusted for factors such as maternal age, education and psychiatric history, neither study adjusted for maternal smoking during pregnancy.

Our study seeks to complement and extend the literature in this area by making use of comprehensive prospective data from the Avon Longitudinal Study of Parents and Children (ALSPAC) (Boyd et al., 2013). Specifically, we assess whether gestational influenza is a risk factor for lifetime hypomania assessed in young adulthood (with and without a history of psychotic experiences, PE), while taking account of a wider range of potential confounding factors than has been considered in previous studies of this research question.

## 2 Methods

### 2.1 Description of cohort and study sample

The ALSPAC birth cohort is comprised of all live births in the County of Avon, UK, with expected due dates between April 1991 and December 1992. The initial cohort consisted of 14,062 live births, with 13,998 alive at one year (http://www.bristol.ac.uk/alspac/). The study website contains details of all the data that are available through a fully searchable data dictionary (http://www.bris.ac.uk/alspac/researchers/data-access/data-dictionary/). Ethical approval for the study was obtained from the ALSPAC Ethics and Law Committee and Local Research Ethics Committees.

From birth, parents completed regular questionnaires about all aspects of their child's health and development. From age 7, children attended assessment centres to participate in tests and interviews on an annual basis. To date, ALSPAC data have been used in a wide range of epidemiological studies in mental health (Fraser et al., 2013, Niarchou et al., 2015). In this study, we assess data on the 2957 ALSPAC participants who completed an assessment of the primary outcome of interest, namely lifetime experience of hypomania, at age 22–23.

### 2.2 Sample selection

From the original ALSPAC cohort, 9359 young adults were invited to complete the *“Your Life Now (at age 21+)”* assessments, which included the HCL-32 questionnaire. Participants could choose from paper or online versions. A total of 3447 participants returned the questionnaire (36.8% response rate), including 2957 with complete answers (representing our study sample).

### 2.3 Outcome measures

#### 2.3.1 Primary outcome: lifetime hypomania assessed in young adulthood

Hypomania was defined using the Hypomania Checklist (HCL-32), assessed when participants were aged 22–23 years. The HCL-32 is a self-completed questionnaire for lifetime experience of manic features (Angst et al., 2005). It asks that people consider a time when they were in a “high or hyper” state and endorse a number of statements about their emotions, thoughts and behaviours at this time. Examples of the 32 symptom statements are: “*I think faster*”; “*I make more jokes or puns when I am talking*”; and “*I take more risks in my daily life*”. The questionnaire also asks about the duration of such episodes and impact on family, social and work life (Carvalho et al., 2014, Court et al., 2014). Although initially developed as a screening instrument for use in people diagnosed with depressive disorders, it is also a sensitive screening tool in non-clinical settings, including samples of young (Meyer et al., 2007, Meyer et al., 2014).

We defined lifetime history of hypomania in line with previous approaches for studies of this nature, namely: a score of 14 or more out of 32 hypomanic features; *plus* at least one response of either “negative consequences” or “negative plus positive consequences”; *plus* a report that these mood changes caused a reaction in others; *plus* a duration of “2–3 days” or more. Overall, this definition of hypomania, which includes severity, impairment and duration criteria, is more conservative than most other studies, which have tended to use only the threshold score of 14 (Hardoy et al., 2005, Meyer et al., 2014). We chose a duration criterion of 2–3 days or more because the 4 day threshold within DSM excludes many individuals with bipolar disorder type II (Goldberg et al., 2009, Pini et al., 2005) and because two days is the modal duration of hypomania for individuals with bipolar II disorder (Angst and Cassano, 2005, Benazzi, 2001). Based on previous work in non-clinical samples, we expected that between 5 and 10% of respondents might satisfy our criteria for hypomania (Holtmann et al., 2009, Meyer et al., 2007).

#### 2.3.2 Secondary outcome: hypomania with previous psychotic experiences (PE)

Hypomania plus previous PE was also studied as an outcome. PE were assessed using the semi-structured Psychosis-Like Symptoms interview (PLIKSi) administered at ages 12 and 18 (Zammit et al., 2009a). The PLIKSi consists of 12 core questions covering hallucinations (visual and auditory); delusions (delusions of being spied on, persecution, thoughts being read, reference, control, grandiose ability and other unspecified delusions); and experiences of thought interference (thought broadcasting, insertion and withdrawal) over the past 6 months. Clinical cross-questioning and probing was used to establish the presence of symptoms, and coding of all items followed the glossary definitions and rating rules for SCAN (Schedule for Clinical Assessment in Neuropsychiatry). PE were coded as present if one or more of the experiences was rated as ‘suspected or definitely present’ by a trained psychologist. Unclear responses after probing were always ‘rated down’, and symptoms only rated as definite when a credible example was provided. In our analysis we included only symptoms that could not be directly attributed to falling asleep/waking or to fever and were reported either in the PLIKSi at age 12 or in the PLIKSi at age 18 (Horwood et al., 2008, Zammit et al., 2013).

#### 2.3.3 Exposure of interest: gestational influenza

Exposure to influenza in utero was based on maternal responses to specific questions about infection which were assessed at three time points: 18 weeks gestation, 32 weeks gestation, and 8 weeks post-partum. Respectively, the questions were: ‘*During this pregnancy so far have you had any of the following (followed by a list of conditions including influenza)?’* (18 weeks gestation)*, ‘In the last 3 months have you had any of the following (again followed by the above mentioned list)?’* (32 weeks gestation) and *‘During the last months of pregnancy (from 7 months onwards) did you experience any of the following (followed by the above mentioned list)?* (8 weeks post-partum). Gestational influenza was coded as ‘yes’ if it was reported at any time during pregnancy, and ‘no’, if it was not reported at any point during pregnancy.

In a further analysis, we looked at exposure to influenza in utero at the three time points separately, i.e., at 18 weeks gestation, 32 weeks gestation, and 8 weeks post-partum (7 months of pregnancy onwards), approximately corresponding to the trimesters of pregnancy.

### 2.4 Confounding variables

We identified *a priori* several potential maternal/paternal and offspring confounding variables based on previous literature in this area (Tsuchiya et al., 2003, Brown and Derkits, 2010, Canetta et al., 2014, Parboosing et al., 2013): parental history of psychiatric problems (maternal or paternal history of depression, schizophrenia, drug addiction, alcoholism and maternal anorexia nervosa or any other maternal psychiatric problem); mother's age at delivery; maternal education level; maternal social class; use of antidepressants during pregnancy (recorded at 18 week and 32 week pregnancy assessments); and tobacco smoking (assessed during the first trimester of pregnancy). Offspring factors included: sex; gestation age at delivery; and birth weight at delivery.

### 2.5 Statistical analyses

Median and interquartile ranges were used to summarise continuous variables, and count and percentages for categorical variables. P-values were obtained using the Kruskal-Wallis and Chi-squared test, and chi square for trend was used for ordinal variable (social class). Univariate and multivariable logistic regression analyses were used to calculate odds ratios (OR) and 95% confidence intervals (95% CI) for hypomania as the dependent variable and with gestational influenza as the independent variable. Multivariable logistic regressions were adjusted for mother's age at delivery, maternal education level, maternal social class, parental history of psychiatric problems, use of antidepressants during pregnancy, maternal smoking during pregnancy, offspring sex, birth weight and gestation at delivery. In a secondary analysis, multinomial logistic regression was used to calculate the OR and 95% CI for exposure to gestational influenza for hypomania with and without previous lifetime experience PE. These regression analyses also adjusted for the confounders listed above.

## 3 Results

### 3.1 Baseline characteristics

Compared to the original cohort, the respondents differed in several characteristics; the respondents were significantly more likely to be female (64.5% versus 43.8%, P<0.001), of a white ethnicity (96.1% versus 94.6%, P=0.001), higher social class (P<0.001), be of higher gestational age at birth [median IQR 40 (39–41) versus 40 (38–41), P<0001], have had a mother with a degree (21.6% versus 9.9%, P<0.001), and mother who reported not having influenza in pregnancy (79.8% vs 75.2%, p<0.001). Respondents were significantly less likely to have mothers that reported ever having depression (6.4% versus 9.9%, P<0.001) and who smoked during pregnancy (15.2% vs 28.4%, P <0.001). They were not significantly more likely to report having PE not attributable to sleep/fever (P=0.091) or to have older mothers at birth (P =0.178).

Overall, 220 (7.4%) respondents satisfied the criteria for hypomania and the remaining participants were classified as a ‘no hypomania’ comparison group (Table 1). The median HCL-32 score was 19 (IQR 16–23) in the hypomania group and 15 (IQR 11–19) in the ‘no hypomania’ group (p<0.001). The two groups did not differ in terms of most of the potential confounding variables (maternal education, maternal depression, social class, maternal age and gestational age) but the hypomanic group were more likely to be male (41.8% versus 34.8%, p<0.035), to have had a mother who smoked during pregnancy (20.9% versus 14.5%, p<0.014) and who took antidepressants during pregnancy (3.05% versus 0.65%, p<0.001) (Table 1).

### 3.2 Gestational influenza and risk of hypomania

Overall, there was little evidence of an association between exposure to influenza in utero and lifetime hypomania assessed in young adulthood: 24.9% of mothers in the hypomania group reported gestational influenza, compared to 20.1% in the comparison group (P=0.121) (Table 1).

### 3.3 Gestational influenza and risk of ‘hypomania with PE’

In a secondary analysis, we tested for an association between gestational influenza and lifetime hypomania with (N=45) and without (N=150) lifetime experience of PE, relative to controls with no history of either hypomania or PEs (N=2088) (Table 2). Twenty five respondents who satisfied criteria for hypomania had incomplete data for psychotic experiences and were excluded. The three groups were similar in terms of most confounding factors (age, gender, maternal education, maternal depression, social class, maternal age and gestational age) but there was a difference in the proportion of mothers who smoked during pregnancy (31.0% in the ‘hypomania plus PE’ group, 16.6% in the ‘hypomania, no PE’ group, and 12.6% in the control group; P=0.001). Use of antidepressants during pregnancy also differed significantly between the three groups (4.8% in the ‘hypomania plus PE’ group, 2.2% in the ‘hypomania no PE’ group, and 0.5% in the control group; P=0.001).

**Fig. 1:**
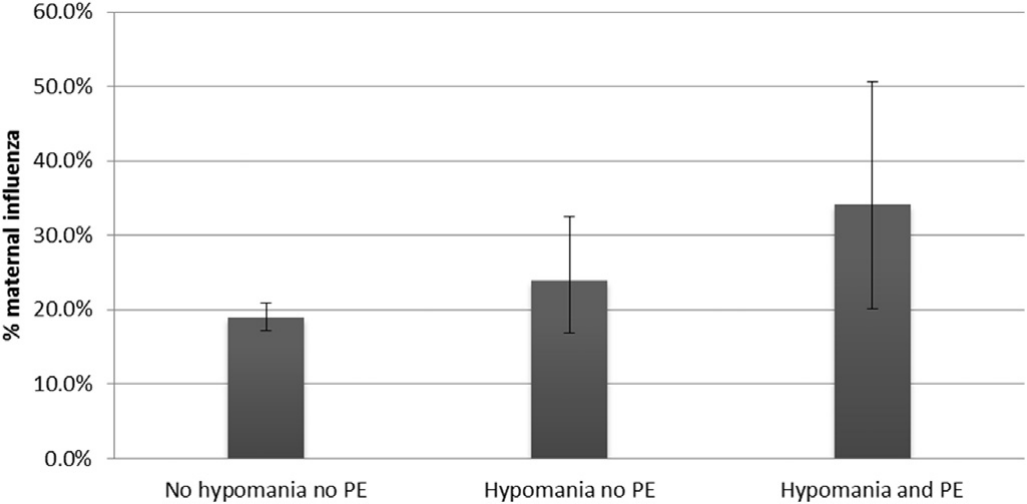
Association between Exposure to Gestational Influenza and Hypomania with and without PE.

There was evidence of a difference in prevalence of exposure to gestational influenza between the groups (34.2% in the ‘hypomania plus PE’ group, 24.0% in the ‘hypomania no PE’ group, and 19.0% in the control group; P=0.025) (Fig. 1 and Table 2). We tested this association further using multinomial linear regression, with the control group as a base group. After adjusting for confounding, there was little evidence that participants exposed to gestational influenza were any more likely to be classified within the ‘hypomania plus PE’ group than in the control group (Table 3a).

Similarly, to test the strength of association between the hypomania groups, we used the ‘hypomania no PE’ as a base group in multinomial linear regression (Table 3b). In the adjusted model participants exposed to influenza in utero were no more likely to be classified within the ‘hypomania plus PE’ group than in the ‘hypomania no PE’ group (Table 3b). Further sub-analyses which divided gestational influenza exposure by trimester did not alter these findings (Table 4a, Table 4b).

## 4 Discussion

In this large prospective birth cohort we found little evidence of an association between exposure to gestational influenza in utero and risk of lifetime hypomania assessed in young adulthood, in line with some previous work in this area (Simanek and Meier, 2015).

Although exposure to gestational influenza was more common for the sub-group of respondents with ‘hypomania plus PE’, this association attenuated after adjusting for confounding factors, with confidence intervals including the null value. Previous work has identified an association between serologically-documented gestational influenza exposure and a sub-group of bipolar disorder with psychotic features (Canetta et al., 2014), although the range of possible confounding factors assessed in that study (including maternal smoking during pregnancy) was limited. It is however difficult to be certain that adjusting for additional confounders within the study by Canetta et al., (2014) would have substantially changed their main finding.

### 4.1 Strengths and limitations

The ALSPAC birth cohort is a large, well-characterised and representative sample of young adults from the UK and our study has the advantage of a prospective design, with relatively large numbers of affected individuals with bipolar disorder, in contrast to some studies (Canetta et al., 2014, Parboosing et al., 2013). Our study also fills an important gap in the literature between clinical and population samples by assessing features of hypomania within a non-clinical cohort of young adults. This work could be considered to represent an advance on previous reports because the level of detail available on the ALSPAC cohort allows for a much wider range of potential confounding factors to be taken into account within analyses, including mother's age at delivery, maternal education level, maternal social class, parental history of psychiatric problems, use of antidepressants during pregnancy, maternal smoking during pregnancy, offspring sex, birth weight and gestation at delivery.

However, we acknowledge a number of limitations. The response rate for those who were sent the HCL-32 questionnaire was 36.8%, which may have resulted in some analyses being under-powered to detect a true effect. There was a selection bias amongst the respondents obvious from the statistically significant differences in some socio-demographic factors and gestational age at delivery. This may have resulted in over-representation of ‘protective’ factors, such as higher education or higher social class within our final sample and may have weakened the association between ‘hypomania’ and exposure to gestational influenza. The under-representation of mothers who reported influenza in the final sample could also have underestimated the effect. Participant attrition has been an issue in studies using more recent outcome measures within ALSPAC and is a potential source of bias in this study. It is however possible that the effect sizes we have observed might be an underestimate because offsprings exposed to influenza in utero (as well as those with bipolar features) were more likely to be missing. Our outcome measure, the HCL-32, may be subject to reporting bias because it relies on self-report in areas such as risk-taking, sexual activity and alcohol use. However, this instrument is well validated as a screening tool for bipolar disorder (Meyer et al., 2014, Vieta et al., 2007). It is also possible that respondents completed the HCL-32 with reference to a period of intoxication with recreational drugs, even though the opening statement specifically asks that they consider “a period when [they] were in a high state, *not related to recreational drug use”.* There have not yet been sensitivity and specificity tests of the HCL-32 as a categorical measure which includes both duration and impact on functioning as criteria but it is likely that by including these features we have improved the sensitivity for a diagnosis of hypomania because previous methods have tended to focus just on a threshold score on the HCL-32, usually 14 out of 32 (Carvalho et al., 2014, Meyer et al., 2007, Vieta et al., 2007). It is also possible that as our study is set in respondents’ young adulthood, larger effect sizes could have been observed if the follow-up was longer.

Another potential limitation relates to the self-reported nature of gestational influenza by mothers. It is possible that some mothers may have been exposed to influenza but did not report this when asked, for example, because of mild symptoms. Clearly serological data on influenza infection during pregnancy would have been preferable but was not available for this study.

## 5 Conclusions

Overall, within a birth cohort followed up into young adulthood, we did not find evidence that a maternal report of influenza infection during pregnancy was a risk factor for lifetime hypomania (or lifetime hypomania plus PE) assessed in young adulthood. Further work in this area should test whether observed associations are likely to be causal or due to confounding in a more rigorous manner than has been done to date. There may also be value in assessing dimensional aspects of psychopathology across the mood disorder-psychosis spectrum, rather than with outcomes defined solely by formal diagnostic categories (such as within ICD-10 and DSM5) (Cuthbert and Insel, 2013, Insel et al., 2010).

## Role of funding source

The ALSPAC is funded by the UK Medical Research Council, the Welcome Trust and the University of Bristol provide core support. This specific analysis was funded by a Strategic Start-Up Grant from the University of Glasgow to DJS.

## Conflict of interest

None. The authors report no conflicts of interest.

## Figures and Tables

**Table 1:** Maternal, pregnancy and offspring characteristics by presence or absence of hypomania in young adulthood.

	**Hypomania N=220**	**No hypomania N=2737**	**P value**
**Maternal characteristics**			
	N, %	N, %	
Age at delivery			
Age>40	2 (1.0)	23 (0.9)	0.912
Age<=40	207 (99.0)	2583 (99.1)	
Missing N=142			
			
Highest educational level			
Degree or above	41 (19.8)	544 (21.4)	0.588
Other qualification	166 (80.2)	1997 (78.6)	
Missing N=209			
			
Social class			
I	21 (11.4)	191 (8.5)	0.473
II	60 (32.4)	825 (36.6)	
III	78 (42.2)	939 (41.7)	
IV	14 (7.6)	131 (5.8)	
V	9 (4.9)	144 (6.4)	
VI	3 (1.6)	23 (1.0)	
Missing N=519			
Parental history of psychiatric problems			
Yes	25 (15.0)	328 (16.0)	0.730
No	142 (85.0)	1724 (84.0)	
Missing N=738			
		
Smoking during pregnancy			
Yes	43 (20.9)	373 (14.5)	**0.014**
No	163 (79.1)	2198 (85.5)	
Missing N=180			
			
**Pregnancy characteristics**			
	Median (IQR)	Median (IQR)	
Gestation at delivery	40 (39–41)	40 (39–41)	0.770
Missing N=142			
	N (%)	N (%)	
Antidepressants during pregnancy			
Yes	6 (3.05)	16 (0.65)	**0.001**
No	191 (96.95)	2436 (99.35)	
Missing N=308			
Gestational influenza			
Yes	46 (24.9)	469 (20.1)	0.121
No	139 (75.1)	1865 (79.9)	
Missing N=438			
			
**Offspring characteristics**			
	Median (IQR)	Median (IQR)	
Age (years)	21.9 (21.5–22.4)	22.01 (21.5–22.4)	0.617
Missing N=26			
	N (%)	N (%)	
Sex			
Female	128 (58.2)	1786 (65.3)	**0.035**
Male	92 (41.8)	951 (34.8)	
Missing N=0			
	Median (IQR)	Median (IQR)	
Birth weight (g)	3380 (3100–3720)	3460 (3120–360)	0.120
Missing N=176			
	Median (IQR)	Median (IQR)	
HCL_32 score	19 (16–23)	15 (11–19)	**0.001**
Missing N=0			
	N (%)	N (%)	
PE (age 12 or 18 years)			
Yes	45 (23.1)	291 (12.2)	**0.001**
No	150 (76.9)	2088 (87.8)	
Missing N=383			

**Table 2:** Maternal, pregnancy and offspring characteristics: hypomania with and without PE, versus controls.

	**Hypomania plus PE N=45**	**Hypomania, no PE N=150**	**Controls (N=2088)**	**P value**
**Maternal characteristics**				
	N (%)	N (%)	N (%)	
Age at delivery				
Age>40	1 (2.3)	0 (0)	17 (0.9)	0.307
Age<=40	42 (97.7)	141 (100)	1964 (99.1)	
Missing N=118				
Highest educational level				
	N (%)	N (%)	N (%)	
Degree or above	9 (20.9)	28 (20.1)	434 (22.4)	0.813
Other qualification	34 (79.1)	111 (79.9)	1506 (77.6)	
Missing N=161				
Social class				
I	3 (7.3)	15 (12.2)	152 (8.75)	0.582
II	14 (34.2)	42 (34.2)	651 (37.5)	
III	16 (39.0)	50 (40.7)	722 (41.5)	
IV	4 (9.8)	9 (7.3)	97 (5.6)	
V	3 (7.3)	6 (4.9)	105 (6.0)	
VI	1 (2.4)	1 (0.8)	11 (0.6)	
Missing N=381				
				
Parental history of psychiatric problems				
Yes	9 (23.1)	13 (11.8)	235 (14.8)	0.235
No	30 (76.9)	97 (88.2)	1352 (85.2)	
Missing N=547				
				
Smoking during pregnancy				
Yes	13 (31.0)	23 (16.6)	246 (12.6)	**0.001**
No	29 (69.1)	116 (83.5)	1713 (87.4)	
Missing N=143				
**Pregnancy characteristics**				
	Median (IQR)	Median (IQR)	Median (IQR)	0.514
Gestation at delivery	40 (39–41)	40 (39–41)	40 (39–41)	
Missing N=118				
Antidepressants during pregnancy	N (%)	N (%)	N (%)	
Yes	2 (4.8)	3 (2.2)	10 (0.5)	**0.001**
No	40 (95.2)	131 (97.8)	1869 (99.5)	
Missing N=228				
	N (%)	N (%)	N (%)	
Gestational influenza				
Yes	14 (34.2)	30 (24.0)	342 (19.0)	**0.025**
No	27 (65.9)	95 (76.0)	1454 (81.0)	
Missing N=321				
**Offspring characteristics**	Median (IQR)	Median (IQR)	Median (IQR)	
Age (years)Missing N=16	21.9 (21.5–22.4)	22.01 (21.5–22.4)	21.9 (21.5–22.4)	0.963
				
	Median (IQR)	Median (IQR)	Median (IQR)	0.181
Birth weight	3390 (3180-3620)	3370 (3080-3700)	3460 (3140-3740)	
Missing N=143				
				
Sex	N (%)	N (%)	N (%)	
Female	29 (64.4)	84 (56.0)	1328 (63.6)	
Male	16 (35.6)	66 (44.0)	760 (36.4)	0.173
Missing=0				
	Median (IQR)	Median (IQR)	Median (IQR)	**0.001**
HCL_32 score	20 (18–23)	19 (16–22)	15 (11–19)	
				

**Table 3a:** Gestational influenza exposure: hypomania with and without PE, relative to controls as a base group (multinomial logit regression)^a^.

**Total N=1459**	**Gestational influenza**	**Univariable**		**Multivariable**	
	**N (%)**	**OR (95% CI)**	**P value**	**OR (95% CI)**	**P value**

Hypomania no PE	Yes=22 (24.2)	1.36 (0.83, 2.24)	0.227	1.36 (0.82, 2.25)	0.231
No=69 (75.8)
Hypomania and PE	Yes=10 (27.8)	1.64 (0.78, 3.45)	0.191	1.50 (0.70, 3.22)	0.299
No=26 (72.2)

a Multivariable model is adjusted for high maternal age, maternal education (degree), maternal social class, use of antidepressants during pregnancy, parental psychiatric history, maternal smoking during pregnancy, offspring sex, birth weight and gestation at birth.

**Table 3b:** Gestational influenza exposure: hypomania with PE and controls, relative to hypomania without PE as a base group (multinomial logit regression)^a^.

**Total N=1459**	**Gestational influenza**	**Univariate**		**Multivariable**	
	**N (%)**	**OR (95% CI)**	**P value**	**OR (95% CI)**	**P value**
Controls (no hypomania no PE)	Yes=253 (19.0)	0.74 (0.45, 1.21)	0.227	0.74 (0.44, 1.22)	0.231
No=1079 (81.0)
Hypomania and PE	Yes=10 (27.8)	1.21 (0.50, 2.89)	0.674	1.10 (0.45, 2.69)	0.831
No=26 (72.2)

a Multivariable model is adjusted for high maternal age, maternal education (degree), maternal social class, use of antidepressants during pregnancy, parental psychiatric history, maternal smoking during pregnancy, offspring sex, birth weight and gestation at birth.

**Table 4a:** Gestational influenza exposure by trimester of exposure: hypomania with and without PE, relative to controls as a base group (multinomial logit regression)^a^.

	**Gestational influenza**	**Univariate**		**Multivariable**	
**Trimester 1 N=1303**	**N (%)**	**OR (95% CI)**	**P value**	**OR (95% CI)**	**P value**
Hypomania no PE	Yes=12 (14.8)	1.66 (0.87, 3.16)	0.122	1.66 (0.86, 3.17)	0.128
No=69 (85.2)
Hypomania and PE	Yes=4 (13.3)	1.47 (0.50, 4.28)	0.481	1.32 (0.44, 3.99)	0.626
No=26 (86.7)
**Trimester 2 N=1236**	**N (%)**	**OR (95% CI)**	**P value**	**OR (95% CI)**	**P value**
Hypomania no PE	Yes=4 (5.5)	1.14 (0.40, 3.23)	0.809	1.15 (0.40, 3.25)	0.794
No=69 (94.5)
Hypomania and PE	Yes=3 (10.3)	2.26 (0.67, 7.71)	0.191	2.57 (0.73, 9.12)	0.144
No=26 (89.7)
**Trimester 3 N=1229**	**N (%)**	**OR (95% CI)**	**P value**	**OR (95% CI)**	**P value**
Hypomania no PE	Yes=6 (8.0)	1.96 (0.81, 4.73)	0.137	1.88 (0.77, 4.60)	0.167
No=69 (92.0)
Hypomania and PE	Yes=1 (3.7)	0.87 (0.12, 6.51)	0.888	0.53 (0.07, 4.24)	0.548
No=26 (96.3)

a Multivariable model is adjusted for high maternal age, maternal education (degree), maternal social class, use of antidepressants during pregnancy, parental psychiatric history, maternal smoking during pregnancy, offspring sex, birth weight and gestation at birth.

**Table 4b:** Gestational influenza exposure by trimester of exposure: hypomania with PE and controls, relative to hypomania without PE as a base group (multinomial logit regression)^a^.

	**Gestational influenza**	**Univariate**		**Multivariable**	
**Trimester 1 N=1303**	**N (%)**	**OR (95% CI)**	**P value**	**OR (95% CI)**	**P value**
Controls (no hypomania no PE)	Yes=113 (9.5)	0.60 (0.32, 1.15)	0.122	0.60 (0.32, 1.16)	0.128
No=1079 (90.5)
Hypomania and PE	Yes=4 (13.3)	0.89 (0.26, 3.00)	0.844	0.80 (0.23, 2.78)	0.720
No=26 (86.7)
**Trimester 2 N=1236**	**N (%)**	**OR (95% CI)**	**P value**	**OR (95% CI)**	**P value**
Controls (no hypomania no PE)	Yes=55 (4.9)	0.88 (0.31, 2.50)	0.809	0.87 (0.30, 2.49)	0.794
No=1079 (95.2)
Hypomania and PE	Yes=3 (10.3)	2.00 (0.42, 9.50)	0.388	2.24 (0.45, 11.10)	0.325
No =26 (89.7)
**Trimester 3 N=1229**	**N (%)**	**OR (95% CI)**	**P value**	**OR (95% CI)**	**P value**
Controls (no hypomania no PE)	Yes=48 (4.3)	0.51 (0.21, 1.24)	0.137	0.53 (0.22, 1.30)	0.167
No=1079 (95.7)
Hypomania and PE	Yes=1 (3.7)	0.44 (0.05, 3.85)	0.460	0.28 (0.03, 2.59)	0.262
No=26 (96.3)

a Multivariable model is adjusted for high maternal age, maternal education (degree), maternal social class, use of antidepressants during pregnancy, parental psychiatric history, maternal smoking during pregnancy, offspring sex, birth weight and gestation at birth.
